# Orientation representation in human visual cortices: contributions of non-visual information and action-related process

**DOI:** 10.3389/fpsyg.2023.1231109

**Published:** 2023-12-01

**Authors:** Thanaphop Threethipthikoon, Zhen Li, Hiroaki Shigemasu

**Affiliations:** ^1^Graduate School of Engineering, Kochi University of Technology, Kochi, Japan; ^2^Guangdong Laboratory of Machine Perception and Intelligent Computing, Shenzhen MSU-BIT University, Shenzhen, China; ^3^Department of Engineering, Shenzhen MSU-BIT University, Shenzhen, China; ^4^School of Information, Kochi University of Technology, Kochi, Japan

**Keywords:** functional magnetic resonance imaging, multivariate pattern analysis, action-related process, orientation, tactile feedback, early visual cortex

## Abstract

Orientation processing in the human brain plays a crucial role in guiding grasping actions toward an object. Remarkably, despite the absence of visual input, the human visual cortex can still process orientation information. Instead of visual input, non-visual information, including tactile and proprioceptive sensory input from the hand and arm, as well as feedback from action-related processes, may contribute to orientation processing. However, the precise mechanisms by which the visual cortices process orientation information in the context of non-visual sensory input and action-related processes remain to be elucidated. Thus, our study examined the orientation representation within the visual cortices by analyzing the blood-oxygenation-level-dependent (BOLD) signals under four action conditions: direct grasp (DG), air grasp (AG), non-grasp (NG), and uninformed grasp (UG). The images of the cylindrical object were shown at +45° or − 45° orientations, corresponding to those of the real object to be grasped with the whole-hand gesture. Participants judged their orientation under all conditions. Grasping was performed without online visual feedback of the hand and object. The purpose of this design was to investigate the visual areas under conditions involving tactile feedback, proprioception, and action-related processes. To address this, a multivariate pattern analysis was used to examine the differences among the cortical patterns of the four action conditions in orientation representation by classification. Overall, significant decoding accuracy over chance level was discovered for the DG; however, during AG, only the early visual areas showed significant accuracy, suggesting that the object’s tactile feedback influences the orientation process in higher visual areas. The NG showed no statistical significance in any area, indicating that without the grasping action, visual input does not contribute to cortical pattern representation. Interestingly, only the dorsal and ventral divisions of the third visual area (V3d and V3v) showed significant decoding accuracy during the UG despite the absence of visual instructions, suggesting that the orientation representation was derived from action-related processes in V3d and visual recognition of object visualization in V3v. The processing of orientation information during non-visually guided grasping of objects relies on other non-visual sources and is specifically divided by the purpose of action or recognition.

## 1 Introduction

Upon grasping an object, the human brain has a remarkable capacity to process information related to its orientation, even when such information is partially or completely obscured from view (Sathian and Zangaladze, 2002; Kilintari et al., 2011). The grasping action onto an object enhances the visual processing of action-relevant features like orientation from the visually guided grasping task (Bekkering and Neggers, 2002; Smith and Soechting, 2005; van Elk et al., 2010; Gutteling et al., 2011). In neuroimaging studies, signal activation during the grasp action has been found in the early visual cortex and the dorsal and ventral pathways (Binkofski et al., 1998; Murata et al., 2000; Culham et al., 2003; van Elk et al., 2010; Gutteling et al., 2015). The activation of the visual areas was further observed during grasping in a dark environment, suggesting that the visuospatial information of an object remains useful for grasping even without online visual input (Singhal et al., 2013; Marangon et al., 2016; Monaco et al., 2017). Specifically, in monkeys, activation was detected in the V3d area when grasping objects in the dark, as previously observed (Kilintari et al., 2011). It has been suggested that the cause of action-related activation in the visual areas is the feedback signals from motor-related areas (Petro et al., 2014). In addition to feedback signals from the motor system, other sources of feedback signals may come from the sensory input when action is taken.

The sensory input from action may be associated with tactile or proprioceptive sensations. Previous studies have shown that orientation discrimination based on tactile sensations involves the visual cortex (Zangaladze et al., 1999; Sathian and Zangaladze, 2002; van der Groen et al., 2013). However, when considering the orientation process from the grasping action, the tactile sensation from touching may be combined with the proprioceptive sensation of the hand position in the peripersonal space. Studies have also demonstrated that hand orientation during reach-to-grasp movements activates the posterior intraparietal sulcus in humans, indicating the involvement of wrist components in object manipulation (Faillenot et al., 1997; Monaco et al., 2011). Furthermore, when pantomime grasping was performed while the eyes were fixated on an object, activation was observed in the anterior intraparietal sulcus area (Króliczak et al., 2007). According to previous studies, tactile and proprioceptive sensations contribute to the orientation process when grasping an object.

On the other hand, apart from sensory input, the feedback signals of action-related processes from the motor system may activate visual information in visual areas. Action-related processes, such as action planning during visuomotor tasks, are found in early visual areas and areas of the intraparietal sulcus (IPS) (Gutteling et al., 2015; Gallivan et al., 2019; Monaco et al., 2020). In addition, action planning in the anterior and posterior parietal cortices modulates the orientation and location of the object during hand alignment and reaching toward an object with a rod-like shape. However, this modulation is only observed in the early visual areas during hand alignment (Velji-Ibrahim et al., 2022). This suggests that action-related processes in visual areas play a role in object-related orientation information.

Despite the extensive research conducted on human visual areas for processing object orientation, the exact mechanisms remain to be elucidated. Without online visual guidance, grasping involves a complex interplay of multiple processes using visual information to process the orientation of an object. To address these gaps, we investigated orientation representation regarding the sensory input and action-related process to understand the factors influencing orientation when online visual guidance is not available. We focused on tactile and proprioceptive sensations as potential non-visual sensory inputs, as well as the processes involved in orientation-related planned and unplanned grasping, to determine their effect on the representation of orientation in visual areas.

**Figure 1:**
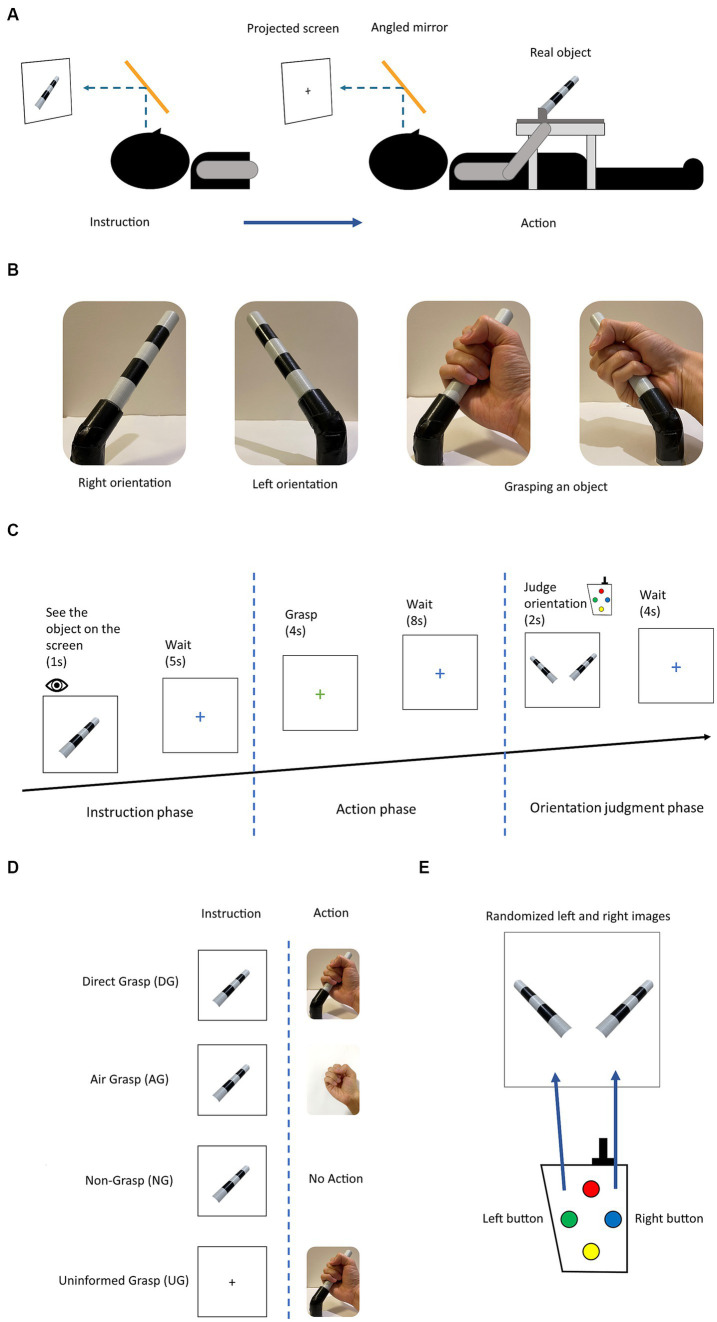
**(A)** Experimental setup: the participant sees the projected stimulus through the angled mirror setup located above the head coil. The photograph of the object was shown for a direct angle presentation of the object in each orientation. **(B)** The object used in the experiment and two orientations with grasping gesture. The object rotated in roll axis of +45° and − 45° by the experimenter. **(C)** Experimental diagram displaying the trial in the experimental session. **(D)** The four action conditions for grasping an object with difference in instruction and action phases. **(E)** The stimuli for button pressing choice in the orientation judgment phase. Participant selected the presents orientation that corresponded to previously grasped or observed object.

Our study aimed to investigate the orientation representation of grasping without online visual guidance in the visual areas. We aimed to determine how representation is affected by input from the non-visual sensory system and signals from action-related processes. The multi-voxel pattern analysis (MVPA) method was used to analyze the blood-oxygenation-level-dependent (BOLD) signals obtained from the fMRI experiments. MVPA was used to decode the pattern differences for conditions in which higher-order visual areas had lower activation signals in the univariate method. The object used in the experiment had an elongated cylindrical shape, which is relevant to the grasping action in the dorsal and ventral visual streams (Fabbri et al., 2016). The object to be grasped was presented in two orientations, which were shown in a random order. Later, in the action phase, participants performed one of the four action conditions designed to integrate the non-visual sensation and action-related processes. The action conditions were as follows: grasping an object after instruction with or without object presence, withholding grasping after instruction, and grasping an object without instruction. The first two conditions integrated proprioceptive information while having a difference in tactile feedback from the presence of the object during grasping. The third condition integrates the action-related processes while withholding the grasp. Finally, the fourth condition involved passive orientation information from proprioceptive and tactile information from the grasping action only, without orientation instruction (Table 1 and Figure 1D). We used the MVPA decoding method to determine the differences in patterns for each action condition. The decoding method was defined as the classification of orientation pairs in each region of interest (ROI) to represent the orientation process of that area. In addition, transfer-type classification or cross-decoding was performed to identify shared patterns across action conditions related to the orientation process. If the results of the transfer classification are statistically significant, it indicates that the area contains a generalization of the sensory-related representation or action-related processes representation. Based on previous research, the early visual and IPS areas were assumed to have high decoding accuracy in the instructed grasp conditions, considering that the planned action can enhance the orientation process (Gutteling et al., 2015). The early visual areas were assumed to be less affected by tactile feedback because visual information is mainly processed in these areas more than somatosensory information. In the dorsal division of the third visual area (V3d), we anticipated a high decoding accuracy during grasping without instruction. We based this assumption on previous findings that demonstrated activation in V3d during the processing of visuospatial information in the dark, in the absence of visual stimuli, suggesting an action-related process (Kilintari et al., 2011). Finally, both the V3d and IPS areas were considered candidates for cross-decoding between grasping with and without instruction from the action-relevant features within the dorsal visual pathway areas (Culham et al., 2003; Singhal et al., 2013).

**Table 1:** The estimate functions of sensory input and action-related processes to each action condition.

	Tactile feedback	Proprioception	Action plan	Visual working memory
Direct grasp (DG)	✔	✔	✔	✔
Air grasp (AG)		✔	✔	✔
Non-grasp (NG)				✔
Uninformed grasp (UG)	✔	✔		

## 2 Materials and methods

### 2.1 Participants

A group of 10 participants (five females, five males; age mean ± SD, 25.94 ± 3.727) were recruited from Kochi University of Technology, Japan, to participate in the fMRI experiments. The selection criteria included having a normal or corrected-to-normal vision and being free from mental illness or neurological disease. Prior to participating, all participants provided written informed consent, in accordance with the Declaration of Helsinki. All participants were compensated for their participation. The study protocol was reviewed and approved by the Human Research Ethics Committee of the Kochi University of Technology.

### 2.2 Experimental design

The fMRI experiment was designed to investigate the effect of the sensory input and motor output on the processing of orientation during grasping with no online visual feedback. The four action conditions were intended to be used for this investigation and were defined as a combination of visual instructions and the task assigned during the action phase. These action conditions were grasping objects after observation (direct grasp condition [DG]), grasping with no object present after observation (air grasp condition [AG]), withholding grasping after observation (non-grasp condition [NG]), and grasping objects without instructions (uninformed grasp [UG]). The DG condition integrated all tactile input and visual instruction, the AG condition had no tactile feedback of the grasping object, the NG condition had action planning but stopped the execution, and lastly, the UG condition had no planning from visual instruction and the participants perceived the orientation of the object from action only (Table 1).

**Figure 2:**
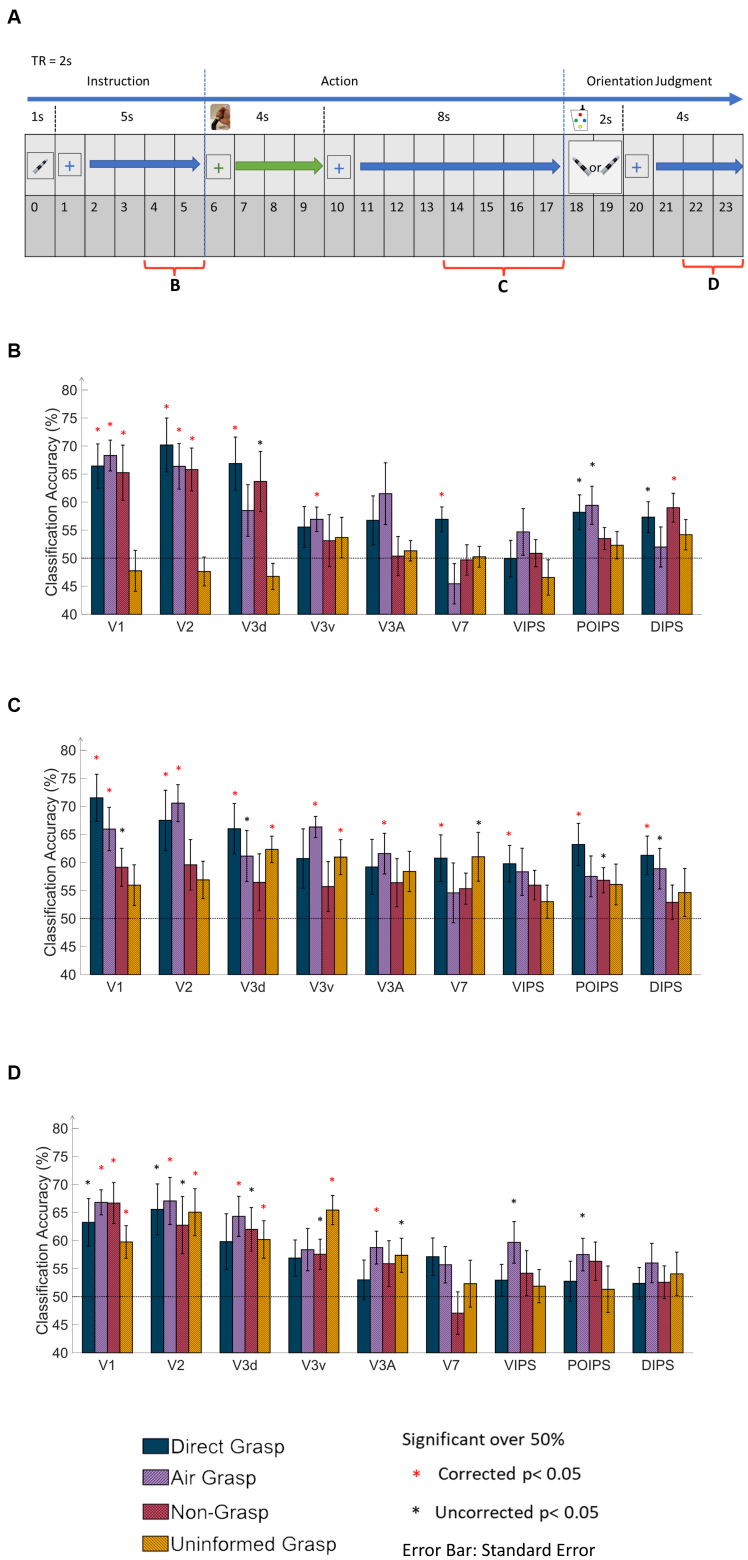
Time windows of the MVPA data and results of same-type classification with ROI-based MVPA. **(A)** The timing diagram of the dataset applied to MVPA classification at 6 s from the onset time in the instruction, action, and orientation judgment phases. The main dataset in the action phase covered 4 s window corresponding to the 4 s of grasping time. **(B)** The bar graph displays results from the MVPA classification in the instruction phase. The results showed high classification accuracy in V1, V2, V3d, and around chance level accuracy from the UG condition. **(C)** The bar graph displays the results in the action phase. The V1 and V2 showed high classification accuracy in DG and AG conditions. Notably, V3d and V3v showed high classification accuracy in the UG condition. **(D)** The bar graph displays results in the orientation judgment phase. The early visual area showed relatively high classification accuracy. In every graph, the results show all conditions of each area by classification (decoding) accuracy and areas. The dashed line indicates the chance level of the classification at 50%. The error bars represent the standard error of the mean across the participants (*n* = 10). The black asterisk represents the statistical significance over the chance level (50%) with two-tailed *t*-tests across the subjects (*p* < 0.05). The red asterisk indicates the statistical significance based on an FDR correction of *q* < 0.05.

In experimental design, each trial comprised of three phases instruction, action, and judgment (Figure 1C). During the instruction phase, participants were presented with the stimulus of an object in two different orientations, either left or right, for a duration of 1 s. Alternatively, for the UG condition, a black fixation cross was presented instead of the object stimulus, for the same duration. Subsequently, the instruction phase was followed by a blue fixation cross signaling a waiting period of 5 s before the commencement of the next phase. The next phase was the action phase, where participants were presented with a green or red fixation cross for a duration of 4 s. The green or red cross, respectively, signaled the participants to either perform a whole-hand grasp or withhold a grasp. All action conditions were performed on the green cross except for the NG condition. The DG condition had a real object for grasping, whereas, in the AG condition, participants made the grasping gesture in the absence of an object. In the NG condition, the participants withheld their grasp during this phase. In the UG condition, participants grasped an object without knowing its orientation. Subsequently, the participants saw the blue fixation cross for a duration of 8 s, waiting for the next phase. During the judgment phase, participants were presented with the stimuli of the object in both orientations, which were randomly ordered. They were prompted to select the correct orientation that they had either previously grasped or observed for a duration of 2 s. Keypads were used for the binary choice of left and right buttons (Figure 1E). Finally, the blue fixation cross was presented again for a duration of 4 s until the commencement of the next trial. A detailed illustration of the trial is shown in Figures 1C, 2A.

All experiments were conducted in a single session. Each session comprised 10 runs, each run consisted of 16 trials, and each trial was further divided into three distinct phases: instruction, action, and judgment (Figure 1C). During the instruction phase, participants were presented with the stimuli of an object. In the subsequent action phase, participants performed an action according to the orientation in the previous phase. Finally, in the judgment phase, participants pressed a button selecting the corresponding orientation (Figure 1C). Each trial had eight possible settings (2 orientations x 4 action conditions) using an event-related design paradigm. The trials were repeated 20 times. All the participants performed 160 trials (2 orientations x 4 action conditions x 20 repetitions). The action conditions used the same timing diagram but with different visual stimuli and actions (Figure 1D).

To calculate the orientation perception performance of each participant, the responses to all judgment tasks per run were recorded. Unanswered assignments were excluded from calculations. All participants were instructed to fixate on the fixation cross at the center of the screen. Any runs with excessive head movements were eliminated. Extraordinary head movement was defined as a head movement greater than 2 mm and/or head rotation greater than 2° from the initial scan of each run. All participants remained still for at least eight echo-planar imaging (EPI) runs. During the session, three participants who were experiencing fatigue took a break in the middle of the session to alleviate their fatigue before continuing with the remaining session.

### 2.3 Stimuli

Participants observed the stimuli through an angled mirror situated above the head coil. The mirror displayed a screen that presented the stimuli from a projector (Figure 1A). The participants’ visual input was from this setting for the entire experiment, without seeing the real object situated in their hip area. An MR-compatible keypad was used to collect judgment choices in the judgment phase. All participants practiced all the action conditions prior to the start of the experiment.

The object for grasping was placed on the table around the participant’s hip area, and the object was fixed on a wooden frame to maintain the position of the correct orientation (Figure 1A). The wooden frame was placed on a plastic table. The keypad was fixed to the right side of a wooden frame on the table. The participants used their right hand to grasp and press the required button.

The experimental stimulus used in this study was a cylindrical object made of plastic and adorned with black and white stripes. The dimensions of the object were 12.5 cm by 1.8 cm in length and diameter. The object was positioned in two different orientations, rotated in a combination of +45° or − 45° on the roll axis (Figure 1B). The photograph of the object was projected on the screen as the visual instruction and eventual choice in the judgment phase. During the instruction phase, the image of the object was presented at the center of the screen. In the judgment phase, two images of the object, each depicting one of the two possible orientations, were presented on the left and right sides of the screen, with a black square at the bottom of both images. The black square changed to a green square when the corresponding button was pressed, indicating the participant’s selection. The order of the images was randomized for each trial.

### 2.4 fMRI data acquisition

All imaging scans were performed using a 3 Tesla Siemens MAGNETOM Prisma MRI scanner at the Brain Communication Research Center of Kochi University of Technology. Participants’ head movements were minimized by securing their heads with MRI-compatible foam pads. Each participant underwent a high-resolution T1-weighted anatomical scan (1 mm^3^), and ROIs were localized and delineated in all separate sessions. During each experimental run, BOLD signals were measured using an EPI sequence with the following parameters: echo time (TE), 58 ms; repetition time (TR), 2,000 ms; 198 volumes per run; 3 mm slice thickness; and interleaved slice acquisition order. The visual, posterior parietal, and posterior temporal cortices were each covered with 34 slices. Additionally, a T2-weighted structural image was acquired for each participant in a 2.5-min run before the corresponding EPI data in one session. The T2-weighted structural data served as reference slices for the motion correction of the EPI data and co-registration between the T1-weighted anatomical images and EPI data in the native anatomical space. Finally, all data were converted to Talairach coordinates.

**Figure 3:**
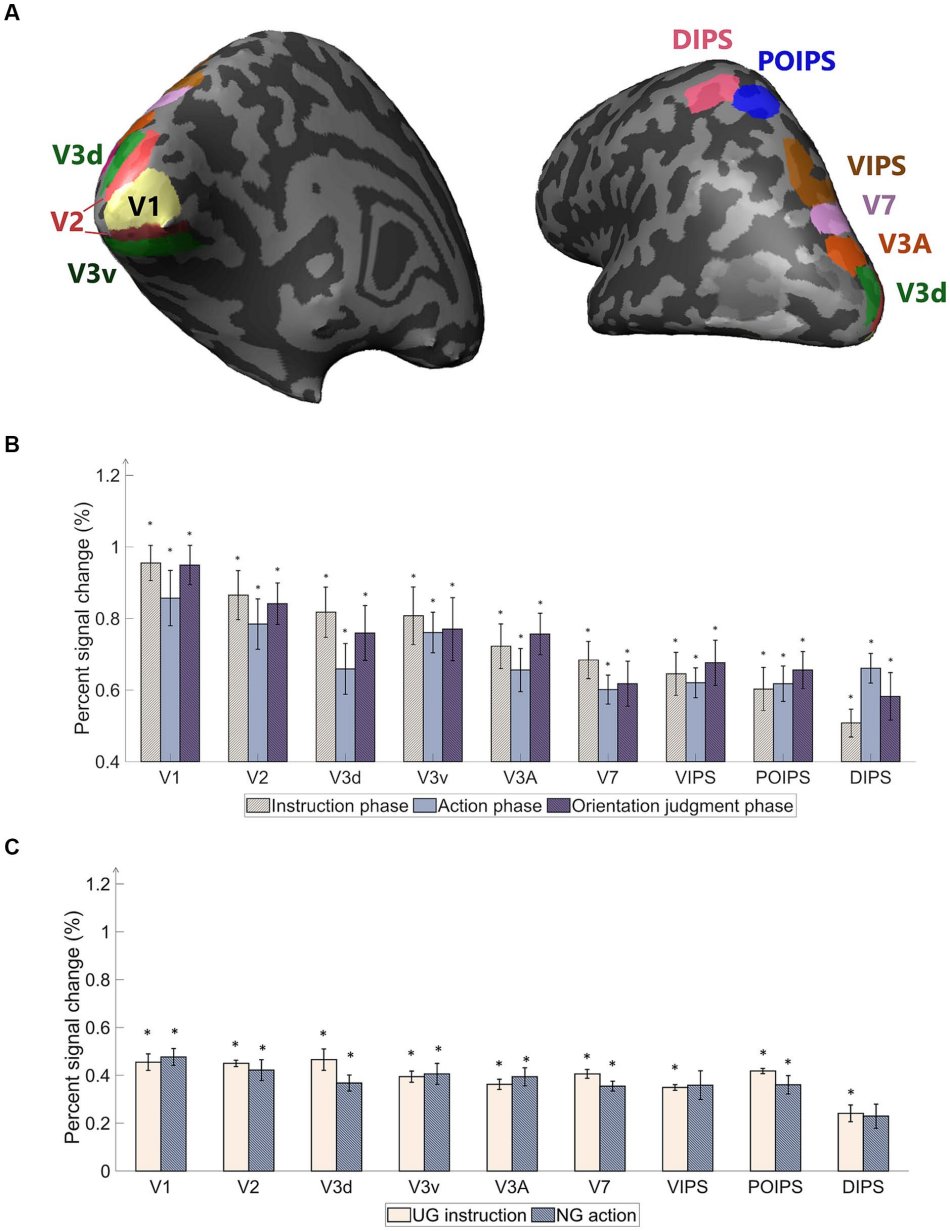
Regions of interest (ROI) used in the experiment and percentage of signal changes in the studied areas. **(A)** The ROIs contain areas in visual cortices. Our study’s early visual cortex (EVC) includes the areas V1, V2, V3d, and V3v. The dorsal areas include V3A and V7. The intraparietal sulcus (IPS) areas comprise the ventral intraparietal sulcus (VIPS), parieto-occipital intraparietal sulcus (POIPS), and dorsal intraparietal sulcus (DIPS). The dark gray pattern indicates the sulci, whereas the light gray pattern indicates the gyri. The ROIs were individually delineated by standard localization sessions (see the fMRI acquisition section). **(B)** Percent signal changes of the areas using averaged BOLD data from instruction, action, and orientation judgment phases. The signal changes decrease toward the higher-order areas. **(C)** Percent signal changes of the areas using averaged BOLD data from UG condition in the instruction phase and NG condition in the action phase. Compared to **(B)** results, the signal changes had the same decreasing tendency toward higher-order areas but lower overall signal strength. The error bars represent the standard error of the mean from all participants (*n* = 10). The asterisk represents the percent signal changes significantly above the 0 from the *t*-test in group data (*p < 0.0005).

The primary objective of our study was to examine the representation of orientation within visual areas under four action conditions in which the object was not visible during grasping (Figure 3A). Following the localizer protocol, retinotopically localized early visual areas (V1, V2, V3d, V3v, and V3A) were individually delineated for each participant using a rotating wedge and expanding ring technique (Sereno et al., 1995; DeYoe et al., 1996; Warnking et al., 2002). Furthermore, V7 was identified as the anterior and dorsal region relative to V3A. In addition to these visual areas, we included regions within the IPS, namely the ventral intraparietal sulcus (VIPS), parieto-occipital intraparietal sulcus (POIPS), and dorsal intraparietal sulcus (DIPS). The IPS areas were identified by comparing the activity of the 3D shapes generated by a rotating motion with that of the 2D shapes generated along a frontoparallel plane (Vanduffel et al., 2002). Additionally, the anterior intraparietal area in nonhuman primates has demonstrated selectivity for the shape, size, and orientation of 3D objects during grasping (Murata et al., 2000). This area in primates has been proposed as a homolog of the human dorsal IPS based on multiple functional tests (Orban, 2016). The signal patterns obtained from each ROI were used to create the classification sample data, with patterns from both the left and right hemispheres merged to represent each ROI. Gutteling et al. (2015) reported no significant differences in contralateral visual areas from the MVPA of visually guided actions toward objects on the left and right sides. In line with this finding, our study opted to include both hemispheres in all analyzes.

## 3 Data analysis

### 3.1 Pre-processing

Data processing and analyzes were conducted using several software packages, including FreeSurfer (Fischl, 2012), BrainVoyager 21 (version 21.0.0.3720, 64-bit; BrainInnovation, Maastricht, Netherlands), and MATLAB R2020b (The MathWorks, Natick, MA, United States). The FreeSurfer software was used to extract white matter (WM) and gray matter (GM) from T1-weighted 3D anatomical images. WM was then employed as a segmentation mask together with GM in BrainVoyager, and the resulting brain was transformed into the Talairach space to generate the cortical surface. Subsequently, the inflated cortical surface was used to define ROIs for MVPA. For the EPI data, 3D motion correction was performed using the T2-weighted image acquired at the beginning of the session without applying spatial smoothing. Co-registration between the EPI data and the T1-weighted image was performed, followed by transformation into the Talairach space.

### 3.2 ROI-based univariate analysis

We performed a univariate analysis of the overall BOLD signal pattern in each ROI to observe the signal changes in each phase, which is expected to decrease in the higher-order visual areas, and we expected that the MVPA can give further distinguishable results. The average BOLD signal pattern was defined as the percentage signal change for stimuli versus baseline, grasping versus baseline, and judgment versus baseline. Additionally, the BOLD signals when no orientation instruction was given (UG condition) and no action was taken (NG condition) were separately analyzed from the average BOLD signal pattern. Three volumes were computed for the hemodynamic latency of the BOLD signal (6 s). For the subsequent analysis, we utilized the mean percentage of signal change across all runs and participants.

### 3.3 ROI-based MVPA

MVPA is a widely recognized analytical approach known for its high sensitivity in detecting differences between conditions. MVPA was applied to the EPI data obtained from each ROI. To conduct MVPA classification, a linear support vector machine (SVM) was employed as a binary classifier in MATLAB. Two classification approaches were employed: same-type and transfer-type. In the same-type classification, both the training and testing of the SVM utilized the same dataset of action conditions to investigate specific cortical patterns related to the orientation of each action condition. Conversely, transfer-type classification involved training and testing the SVM with different datasets across the action conditions, such as training with the DG condition and then testing with the UG condition, and vice versa. The results obtained from the transfer classification were used to assess the common patterns shared across the different action conditions. For each classification, pairs of distinct orientations were used as inputs for the SVM to determine the accuracy of the orientation classification. The classification accuracy was defined as the orientation classification of the respective ROI.

For each ROI, the selection of the ROI from both hemispheres involved choosing the top 250 voxels with contrast for the stimulus versus the fixation baseline. In cases where the ROI contained fewer than 250 voxels, all available voxels were included. In the instruction and judgment phases, a single volume scan was calculated after a 4-s interval. In the action phase, the average values of two volumes were calculated, taken 8 s after the onset, to account for the longer movement time of 4 s (Figure 2A). These differences were transformed into z-scores and used to train and test the SVM.

The leave-one-run-out method was employed to evaluate the performance of MVPA classification. Data from one run was used for testing, while data from the other runs served as the training data. This procedure was repeated for all runs and the accuracy of each iteration was averaged to determine the accuracy of the participant. Subsequently, the classification accuracies across participants were averaged for each ROI.

To determine the statistical significance of the MVPA results, a two-tailed one-sample t-test was performed across participants, with a chance level of decoding set at 50% for each ROI with a value of p threshold of 0.05. To correct for multiple comparisons (number of ROIs x number of tests) across the nine ROIs, the false discovery rate method (FDR) was applied, with an adjusted value of p threshold of 0.05 (Benjamini and Yekutieli, 2001).

## 4 Results

### 4.1 ROI-based univariate analysis

A univariate analysis was conducted to ensure that the BOLD signals change occurred throughout the phases. The primary “percent signal changes” results from stimuli versus baseline, grasping versus baseline, and judgment versus baseline were high in the early visual areas, while the signals decreased toward the higher dorsal areas (Figure 3B). Although cortical activity was detected, the signal changes were weak along the higher-order visual areas (Figure 3B). The percent signal changes were significantly high (above 0) (*p* < 0.0005). A statistically significant difference among the three contrasts was not found. The percentage signal changes of UG instruction versus baseline and NG action versus baseline were lower than the average signals of all conditions (Figure 3C). DIPS showed no significance in NG action versus baseline. This may be due to the absence of retinal information of the object input in the visual areas and the absence of action performed as in the other three conditions. The results suggest further investigation using MVPA classification methods to assess the characteristics of each condition in each phase.

### 4.2 ROI-based MVPA results

#### 4.2.1 Same-type classification

The same-type classification or decoding refers to the ability to classify the orientational difference of observed or grasped object in each visual area. If the accuracy is significant then the orientation is represented in that area. The classification process involved analyzing the BOLD signal from each ROI within the visual cortices, which was divided into three phases: instruction, action, and orientation judgment (Figure 2A). In the instruction phase (Figure 2B), the DG, AG, and NG conditions exhibited high decoding accuracy in V1 and V2; whereas, the UG condition displayed decoding accuracy close to chance levels across all areas. This observation may be attributed to the lack of visual instruction in the UG condition.

The action phase classification showed the ability to classify the orientation difference during grasping action or withhold grasping. In the action phase (Figure 2C), the DG condition demonstrated significantly higher accuracy in most areas, except V3v and V3A. The AG condition exhibited significantly high accuracy for V1, V2, V3v, and V3A, along with a relatively high accuracy for V3d. The NG condition displays no significant decoding accuracy for any area. In contrast, the UG condition showed significantly higher accuracy for V3d and V3v and relatively higher accuracy for V7. There are four points to be stated from these results. First, both the visual instruction and execution of the action itself showed the main contribution to decoding accuracy in V1 and V2. Second, the V3 area showed different decoding results depending on the tactile feedback of the object. Thirdly, in the V7 and IPS areas, the tactile feedback from the action affected the decoding results the most. Fourth, the withhold action (NG condition) showed that the decoded cortical pattern was affected in all areas. This suggested that the decoding results in this phase represented action-related orientation.

In the judgment phase (Figure 2D), V1, V2, and V3d demonstrated high accuracy, whereas the IPS areas showed non-significant accuracy in most conditions. Specifically, areas V1, V2, V3d, and V3A exhibited significant accuracy under the AG conditions. Finally, V3v displayed significantly high accuracy in the UG condition. This may indicate that decoding results were the orientation-related cortical pattern from visual image cues during judgment, action of button pressing, and evoke working memory from orientation in previous phases.

#### 4.2.2 Transfer-type classification

The results of the transfer-type classification are presented in the same order as those of the same-type classification. In the instruction phase, we conducted transfer-type classifications using the UG and other conditions as follows: “DG & UG,” “AG & UG,” and “NG & UG.” These transfer-type classifications aimed to identify common patterns associated with action preparation that may not be orientation-specific because of the absence of instructions in the UG condition. The decoded results from the selected pairs of transfer-type classifications in the instruction phase were close to the chance level. We did not perform transfer-type classification among conditions with the same stimuli instructions (DG, AG, and NG) to avoid decoding common visual cues orientation patterns.

**Figure 4:**
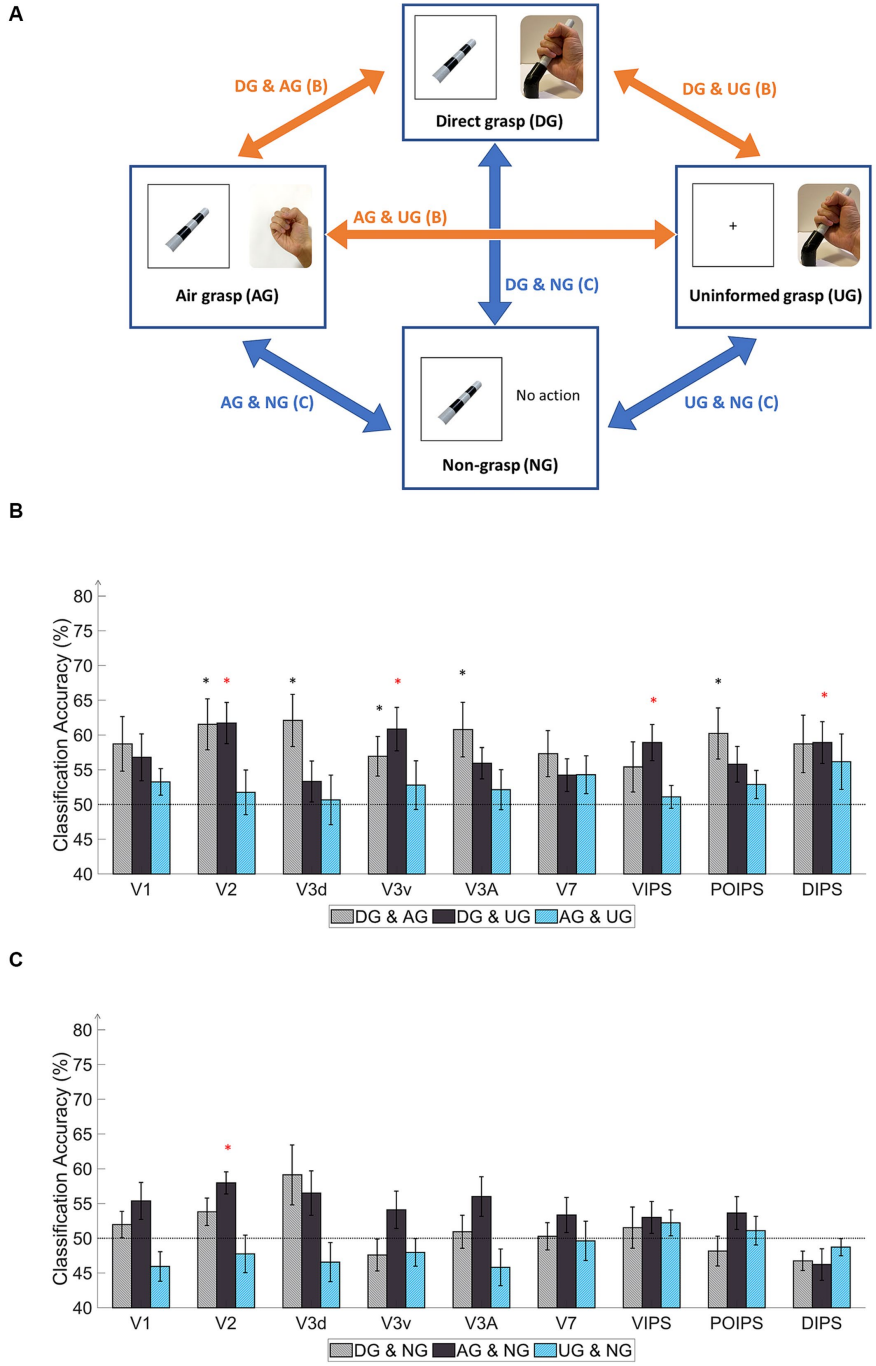
Schematics and results of ROI-based MVPA “transfer-type” orientation classification schematics in the action phase. **(A)** The schematic indicates transfer-type orientation classifications. The transfer-type orientation classification is displayed by arrows pointing to the pair of conditions to transfer as follows: DG & AG, DG & UG, AG & UG, AG & NG, DG & NG, and UN & NG. **(B)** The bar graph indicates the decoding results from the MVPA classification in the transfer-type classification of the DG & AG, DG & UG, and AG & UG. The areas V2, V3v, VIPS, and DIPS showed significantly high classification accuracy in “DG & UG.” **(C)** The bar graph displays decoding results from the transfer-type classification of AG & NG, DG & NG, and UN & NG. Only V2 showed significantly high decoding accuracy in “AG & NG.” In every graph, the dashed line indicates the chance level of the classification at 50%. The error bars represent the standard error of the mean across the participants (*n* = 10). The black asterisk represents the statistical significance over the chance level (50%) or between the grasping types in (2) with two-tailed *t*-tests across the subjects (*p* < 0.05). The red asterisk indicates the statistical significance based on an FDR correction of *q* < 0.05.

Six transfer-type classifications were used in the action phase (Figure 4A). In Table 1, we identified the estimated functions regarding feedback of sensory input and motor system; whereas, when transfer classification was performed across conditions, we could determine what functions can be decoded. When all functions were included in the DG condition, the other conditions were similar and lacked some functions. The transfer-type classification across the DG and AG conditions revealed a common pattern related to proprioception, action planning, and possibly visual working memory regardless of tactile feedback from the object (Table 1). The transfer-type classification of both DG to NG and AG to NG might reflect the visual memory of the object related to the imagination of the object orientation. The common pattern observed in the DG and UG conditions indicates the common involvement of tactile feedback and proprioception. Finally, the transfer classification between the AG and UG conditions may be related to proprioception.

Regarding the transfer-type classification results in the action phase (Figure 4B), the “DG & AG” transfer classification showed relatively high accuracy for V2, V3d, V3v, V3A, and POIPS. The decoded pattern from these areas may have the potential for proprioception, action planning, and visual working memory but some underlying tactile feedback patterns may not be common in the processing of orientation. The “DG & UG” transfer classification demonstrated significantly high accuracy for V2, V3v, VIPS, and DIPS. These areas had a common cortical pattern regarding tactile sensation and proprioception. Only V2 showed significantly high accuracy in the “AG & NG” transfer classification (Figure 4C). This area may involve visual working memory. Conversely, no significant results were found in the transfer-type classification of the “DG & NG,” “AG & UG,” or “NG & UG” conditions. These transfer classifications might not have enough common cortical patterns across conditions.

In the judgment phase, the transfer-type classification results indicated a common orientation-related pattern when selecting stimuli using buttons across action conditions. Overall, the early visual areas displayed high accuracy. The “DG & AG” transfer-type classification showed significance for V1, V2, V3d, V3A, and VIPS. Relatively high accuracy was observed in V1, V2, and V3A for the DG-to-AG conditions. The “DG & UG” conditions exhibited high accuracy for V2, V3d, and V3A. “AG & NG” transfer classification displayed high accuracy for V1, V2, V3d, and V3v. The “AG & UG” transfer classification showed significantly higher accuracy for V1, V2, and V3v. Finally, the “NG & UG” conditions yielded significant results for V1, V2, and V3d, and relatively high accuracy for V3A. Overall, the transfer classification of the judgment phase suggested the cortical pattern regarding the judgment on orientation in early visual areas and VIPS.

In addition, we additionally perform across-phase transfer classification where we perform training the data in the vision phase then test in the action phase and vice versa. This procedure was performed to check whether the orientation representation was shared across the vision and action phases. The decoding results showed significantly low accuracy below the chance level for the DG in V1, V2, and V3d. The AG condition showed significantly low accuracy below the chance level in V1, V2, V3d, and V3v, and NG conditions had significantly low accuracy below the chance level in V1, V2, and V3d areas. The cross-phase decoding results showed that the decoded representation is not same across vision and action phase but not entirely unrelated. This suggested some counter or inverse relationship between the orientation process in the retinotopic (vision phase) and action (action phase) representations (see the Supplementary material for all transfer-type classification results).

## 5 Discussion

Using fMRI data and the MVPA method, this study aimed to examine the representation of orientation under four non-visually guided action conditions, to understand the factors among the tactile, proprioception, and action-related processes that affect the orientation process in the visual cortices. In the same-type classification (Figure 2C), the DG condition demonstrated a high decoding accuracy for V1, V2, V3d, V7, VIPS, POIPS, and DIPS. In the AG condition, significant decoding accuracy was observed in V1, V2, V3v, and V3A. Notably, in the NG condition, in which no action execution took place, although visual instructions were given, the results showed non-significant overall accuracy. This suggests that cortical representation of orientation relies on the action itself in the action phase. Particularly, in the UG condition, the orientation cortical pattern could be decoded from V3d and V3v, which was a surprising result and suggested the involvement of other sources of information from somatosensory and motor systems in the orientation process.

A further distinction of results emerged between DG and AG conditions, particularly in higher dorsal regions (V7 and IPS areas), underscoring the impact of tactile feedback and action-related processes. Alternatively, the cross-decoding results from the V3v and DIPS revealed a shared pattern across the DG-UG condition, whereas the V3d did not exhibit significant transfer decoding. This shared pattern may involve additional cognitive processes, such as visualization of the object during grasping from tactile feedback and proprioception in the visual cortices.

### 5.1 V1 and V2 mainly process orientation from visual information

Orientation representations in the V1 and V2 areas exhibited a similar tendency, with significantly high decoding accuracy under both the DG and AG conditions (Figure 2C). These conditions involved visual object instruction and the execution of the grasping action, with proprioceptive information as a common input. The absence of tactile feedback under the AG condition had a relatively minor impact on the decoded orientation patterns in V1 and V2. This can be attributed to the enhancement of the decoded cortical pattern related to object orientation perception by action planning and execution of the grasping action (Gutteling et al., 2015). Furthermore, V1 and V2 may incorporate a visual working memory component (Harrison and Tong, 2009). Visual information stored in memory can be reactivated during action performance when no real-time visual feedback is available (Singhal et al., 2013; Monaco et al., 2017). Consequently, non-visual information, including tactile feedback, may have less relevance in affecting orientation representation in V1 and V2. In contrast, the NG condition, in which no action occurred, exhibited low decoding accuracy, indicating that the contribution of action-related processes is important to the orientation process in the action phase, especially with no online visual feedback of action. Moreover, the UG condition showed low decoding accuracy in V1 and V2, suggesting that prior information about object orientation is important for the planning and processing orientation during grasping with no real-time visual feedback. Additional results from cross-phase decoding analysis (vision to action and vice versa) revealed a potential inverse-orientation cortical pattern that was correlated across different phases in the DG, AG, and NG conditions. This suggests a distinct representation of visual-related orientation information that was transformed during action execution. Therefore, visual instruction and action execution are both necessary for the cortical pattern of orientation representation in V1 and V2 from evidence in NG and UG results. Overall, our findings suggest that orientation processing in V1 and V2 relies primarily on visually related information evoked by the action process.

### 5.2 Different functional processes between V3d and V3v

In the case of the V3 areas, the orientation representation exhibited distinct functional differences between the dorsal and ventral sections. Specifically, V3d displayed reliance on tactile feedback, as evidenced by the significant decoding results under the DG condition and non-significant results under the AG condition. Previous studies on macaque monkeys emphasize the roles of V3 and VIP in visuotactile integration (Négyessy et al., 2006). Additionally, the V3d is activated during object grasping in the absence of visual stimuli (Kilintari et al., 2011), further supporting the involvement of tactile feedback and action-related processes in this region. Moreover, previous studies have consistently demonstrated selective activation of the lower visual field within the peripersonal space (Previc, 1990; Danckert and Goodale, 2001, 2003), confirming that the V3d area is intricately linked to action-related processes. Our study’s characteristic findings in V3d strongly suggested the integration of signals from the somatosensory and motor systems in the representation of orientation during grasping.

Contrarily, V3v, situated in the ventral pathway, is primarily associated with visual recognition processes (Mishkin et al., 1983; Goodale and Milner, 1992). In contrast to V3d, V3v demonstrated significant decoding results under the AG condition, but non-significant results in the DG condition. During grasping in the dark, the activation of the ventral stream area has been observed and is suggested to be related to the visual object recognition process (Singhal et al., 2013). The absence of tactile feedback during object grasping may stimulate visual imagery to compensate for missing information, thereby triggering the visual recognition process, and significantly affecting the decoding accuracy. Furthermore, the visual imagery observed in V3v was action-triggered, which explains the low decoding accuracy observed under the NG condition.

In the UG condition, orientation perception relied solely on uninformed grasping, without visual or memory-related information. Therefore, action planning and the use of memory components for objects were not assumed to be presented. Notably, both V3d and V3v demonstrated significant decoding accuracy under these conditions. In V3d, orientation information is likely utilized for specific action-related processes for each action of the DG and UG, resulting in different orientation patterns when the orientation is not pre-informed in the UG. The action of grasping objects in the dark can activate cortical patterns in the V3d (Kilintari et al., 2011). Moreover, the lower visual field is selectively active for action in the peripersonal space (Previc, 1990; Danckert and Goodale, 2001, 2003), suggesting an action-related process in V3d. The transfer-type classification results for V3d indicate the absence of a shared pattern between the informed (DG) and uninformed (UG) orientation conditions. This suggested a different action process in V3d when an action was not planned.

In V3v, although the DG condition showed non-significant results, the decoding results of the transfer-type classification of the DG and UG were significant. This suggests that the classifier in the UG condition has a major influence on the cross-decoded results. During grasping, the visual recognition processes in the V3v may involve object visualization and integration of tactile feedback to compensate for missing visual instructions in the UG condition. This notion is supported by studies showing that the ventral visual areas can process object shape recognition, even with partial perception through a narrow slit (Orlov and Zohary, 2018), supporting the object visualization aspect of the V3v in our study. Overall, these findings emphasize the differential orientation processing in the dorsal and ventral pathways within the V3 areas.

### 5.3 Higher dorsal areas representation and contribution of NG condition

In the higher dorsal areas, namely V7, VIPS, POIPS, and DIPS, the representation of orientation appears to rely on tactile feedback, albeit for different reasons compared to V3d, primarily because of the involvement of visuomotor processes (Culham et al., 2003; van Elk et al., 2010). Dorsal pathway areas in the parietal lobe are close to the somatosensory cortex, which receives feedback signals related to motor activity. Notably, a previous study demonstrated the integration of visual and tactile signals of the hand in the anterior IPS in humans (Gentile et al., 2011). During grasping actions, tactile signals may exert a greater influence on the visuomotor processes, particularly when real-time visual feedback is unavailable.

During the action phase, three action conditions activated action-related processes, which are characterized by feedback signals from motor-related areas associated with visuomotor tasks such as grasping (Culham et al., 2003; Petro et al., 2014; Gutteling et al., 2015; Gallivan et al., 2019). In AG and DG conditions, the grasping was planned, while in the UG condition, unplanned grasp was performed. These three conditions showed significant classification accuracy in certain areas. In contrast, the NG condition, where no grasping action was performed, resulted in low classification accuracy across all areas. On the other hand, action planning involves a memory component, specifically working memory (Fiehler et al., 2011; Schenk and Hesse, 2018). Furthermore, a perspective review by van Ede (2020) has proposed that the action modulates visual working memory bidirectionally in visual cortices. These previous studies suggested that in the NG condition, although the object’s mental image may have been retained, the memory component does not contribute significantly to the cortical pattern when the grasping action and online visual input are absent.

### 5.4 Limitations

The AG condition in our study had certain limitations that should be acknowledged. Firstly, the difference between the AG condition and other grasping conditions may extend beyond the absence of tactile sensation. The performance of grasping action in the air, without physical contact with an object, may not fully replicate real-world grasping scenarios. In essence, the AG condition represented a form of “pantomime grasping.” Previous investigations comparing pantomime and real grasping tasks have shown that real grasping elicits greater activation in parietal areas (Króliczak et al., 2007), suggesting distinct action processes that could result in the decoding of orientation in our study. Furthermore, attention during the initial stages of grasping an object may affect the grasping action. The DG and UG conditions require careful alignment and shaping of the hand around the object, while the AG condition may involve less attention to this aspect. The less attention to hand position could affect the cortical pattern. Future research on non-visually guided actions should address effective control of tactile feedback presence or absence from objects, as well as ensuring control over the participant’s attention during the trials.

The content of visual memory in the NG condition suggested a limitation of this study. Participants had accurately judged orientation in a later phase indicating the retention of instructed orientation in memory despite low decoding accuracy in the action phase. The memory content may take the form of semantics (e.g., left, or right) rather than a visual image of the object, serving as cues for button selection preparation. A recent study by Davis et al. (2021) demonstrated that semantic representations of objects can predict perceptual memory in visual cortices. In particular, semantic representations of the object (“orange”) were derived from normatively observed (“is round”), taxonomic (“is a fruit”), and encyclopedic (“is sweet”) characteristics. In our study, the low decoding accuracy for “left” or “right” orientation representation suggested a different semantic process for orientation information. To address this limitation, future studies could explore alternative designs or include control conditions to investigate the specific contribution of memory and semantics in orientation processing.

The selection of the ROI may have certain limitations. Initially, our focus was on the early visual and dorsal pathways, given their association with the action process (Culham et al., 2003; Petro et al., 2014). However, our study revealed the involvement of the ventral area V3v in visual recognition processes during orientation representation in grasping tasks without visual feedback. This finding highlights the potential for investigating orientation processing in higher ventral areas that are responsible for object recognition during action in the absence of visual feedback.

## 6 Conclusion

In conclusion, our study revealed that non-visual information, including tactile feedback, proprioceptive information, and action-related processes, plays a significant role in orientation representation within the human visual cortex. Orientation representation within the V1 and V2 exhibited a strong dependence on both the visual information and the action process. In the V3 areas, the findings highlighted differential processing in the dorsal and ventral sections of V3, where tactile feedback influenced orientation perception in V3d for the specific action-related process, while visual imagery of the object compensated for the absence of object information in V3v. These findings provide valuable insights into the complex interplay between sensory inputs and motor-related processes, and their impact on orientation perception, highlighting the variability among different areas within the human visual cortex.

## Data availability statement

The original contributions presented in the study are included in the article/Supplementary material, further inquiries can be directed to the corresponding author.

## Ethics statement

The studies involving humans were approved by Human Research Ethics Committee of the Kochi University of Technology. The studies were conducted in accordance with the local legislation and institutional requirements. The participants provided their written informed consent to participate in this study.

## Author contributions

TT and HS designed the study and collected the data. TT contributed to the data analysis and wrote the manuscript. ZL provided in-house MATLAB scripts for the analysis and additional technical support. HS supervised data analysis and revised the manuscript. All authors contributed to the article and approved the submitted version.
